# The emerging roles of irisin in vascular calcification

**DOI:** 10.3389/fendo.2024.1337995

**Published:** 2024-02-09

**Authors:** Shuangshuang Wang, Siwang Hu, Yuping Pan

**Affiliations:** ^1^ Department of Cardiology, The First People’s Hospital of Wenling (The Affiliated Wenling Hospital of Wenzhou Medical University), Wenling, Zhejiang, China; ^2^ The Orthopaedic Center, The First People’s Hospital of Wenling (The Affiliated Wenling Hospital of Wenzhou Medical University), Wenling, Zhejiang, China; ^3^ Department of Internal Medicine, Yuhuan Second People’s Hospital, Yuhuan, Zhejiang, China

**Keywords:** irisin, vascular calcification, risk factors, predictor, protective role

## Abstract

Vascular calcification is a common accompanying pathological change in many chronic diseases, which is caused by calcium deposition in the blood vessel wall and leads to abnormal blood vessel function. With the progress of medical technology, the diagnosis rate of vascular calcification has explosively increased. However, due to its mechanism’s complexity, no effective drug can relieve or even reverse vascular calcification. Irisin is a myogenic cytokine regulating adipose tissue browning, energy metabolism, glucose metabolism, and other physiological processes. Previous studies have shown that irisin could serve as a predictor for vascular calcification, and protect against hypertension, diabetes, chronic kidney disease, and other risk factors for vascular calcification. In terms of mechanism, it improves vascular endothelial dysfunction and phenotypic transformation of vascular smooth muscle cells. All the above evidence suggests that irisin plays a predictive and protective role in vascular calcification. In this review, we summarize the association of irisin to the related risk factors for vascular calcification and mainly explore the role of irisin in vascular calcification.

## Introduction

Vascular calcification is a common complication of many diseases characterized by calcium deposition on the vessel wall, including diabetes, atherosclerosis, chronic kidney disease (CKD), and hypertension (1). Abnormal vascular calcification might impair vasomotor function and increase the risk of plaque rupture (2). The cohort studies showed that coronary artery calcification was independently associated with major adverse cardiovascular events (MACE) and was even the leading cause of death in CKD patients (3, 4). Due to the complexity of its mechanism, unfortunately, there is currently no effective drug to relieve vascular calcification. Therefore, exploring the mechanism of occurrence and development of vascular calcification and effective therapeutic targets is imperative.

Irisin, first reported in 2012 by Boström et al, is a 112-amino acid myogenic cytokine produced from the shed extracellular region of the transmembrane protein Fibronectin Type III Domain-Containing Protein 5 (FNDC5) (5). In the skeletal muscle cells, exercise could activate PGC-1α, thereby promoting the expression of FNDC5, which is processed into irisin (5). Interestingly, the translation initiation codon of human FNDC5 is atypical ATA rather than typical ATG (6). While the ATA codon in human FNDC5 may represent a null mutation, humans were presumed incapable of producing irisin (7, 8). The key findings using modified mass spectrometry techniques confirmed that irisin was real and expressed mainly at the atypical ATA start codon of FNDC5 (9). Subsequently, irisin has been shown to bind to its receptor αV/β5 integrin, thereby playing a protective role in bone remodeling and gut barrier function (10–12). Furthermore, Mu et al. found that the extracellular chaperone heat shock protein 90α (HSP90α) was involved in irisin-mediated integrin activation in mice (13). Exercise can lead to an increase in irisin levels, as well as the release of HSP90α. Then, HSP90α could promote αVβ5 activation, enabling irisin to act efficiently through its integrin receptor.

Subsequent research confirmed that exercise with the proper intensity and duration could stimulate the release of irisin, sequentially regulating glucose homeostasis, energy metabolism, and white adipose tissue browning (14–16). A meta-analysis containing 921 participants showed that exercise training could significantly increase irisin and decline insulin, glucose, and insulin resistance (17). Also, high-fat diets could increase the expression of Zfp57 by inhibiting the AMPK pathway, thereby inducing the inhibition of FNDC5 and further exacerbating insulin resistance in mouse muscle cells (18). The available evidence suggests that irisin takes part in a variety of metabolic processes and has protective effects against many metabolic diseases, such as diabetes, metabolic syndrome, obesity, etc. (19).

In addition to skeletal muscle, cardiac muscle is another source of irisin (20). As a myotropin, irisin is an important target for cardiac exercise rehabilitation, which can improve cardiac function after myocardial infarction (21). Irisin may alleviate ischemia-reperfusion injury by reducing oxidative stress, improving mitochondrial health, and inhibiting inflammation (22). Collectively, increasing evidence confirms the emerging protective effects of irisin in several cardiovascular diseases (23). In this review, we mainly focused on the role of irisin in risk factors and vascular calcification and provided useful clues for the application of irisin to relieve vascular calcification.

## Irisin modulates risk factors for vascular calcification

**Figure 1:**
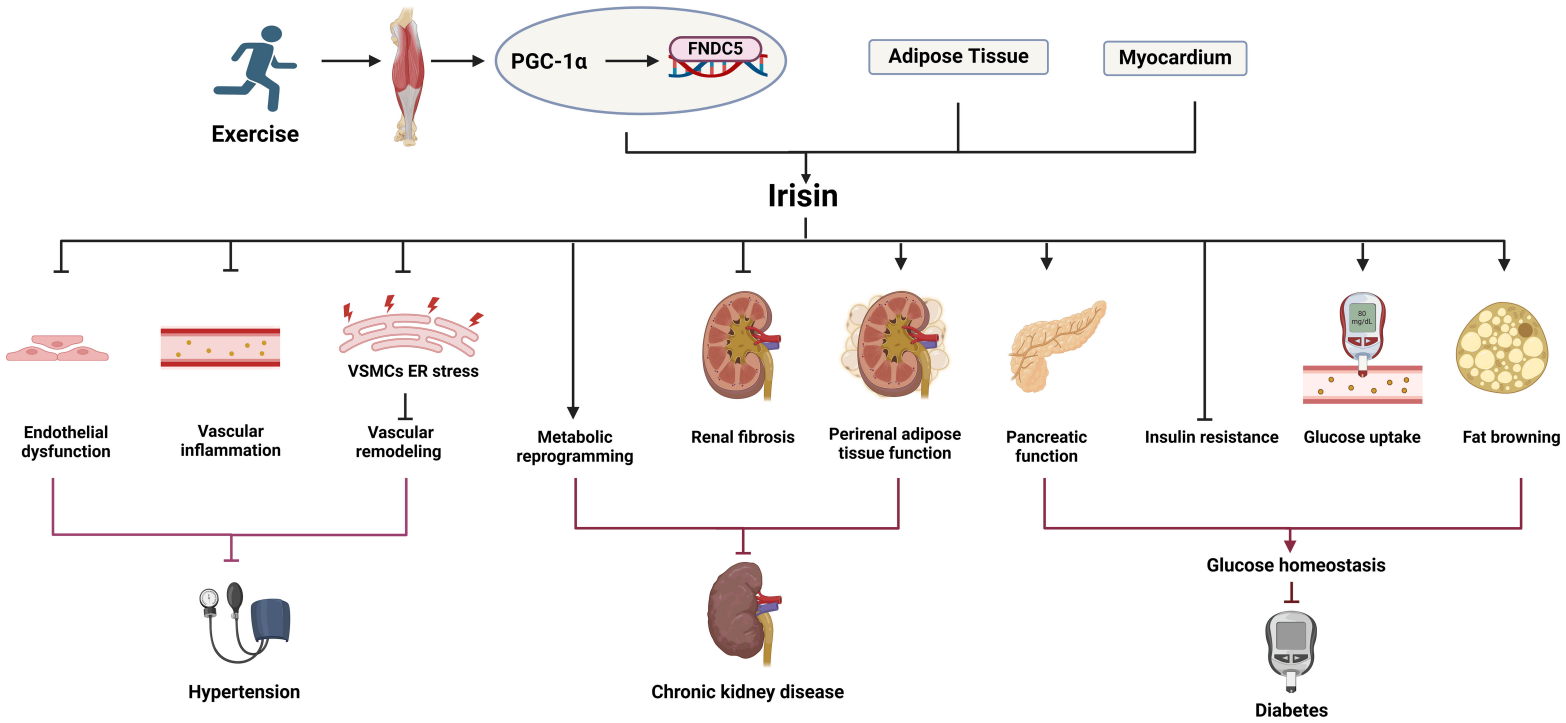
The role of irisin in the risk factors for vascular calcification. Irisin can inhibit hypertension, diabetes, chronic kidney disease, oxidative stress, inflammation and other risk factors related to vascular calcification.

Vascular calcification was previously thought to be a passive degenerative disease with aging. With a deeper understanding of molecular mechanisms, vascular calcification is defined as an active, reversible, and highly regulatable process affected by many factors (24). Currently recognized risk factors for vascular calcification include genetic factors, hypertension, diabetes, lipid metabolism disorders, inflammation, oxidative stress, imbalance of calcium and phosphorus homeostasis. Next, we mainly discuss the role of irisin in suppressing risk factors for vascular calcification (**Figure 1**).

### Irisin and hypertension

Hypertension is a common chronic condition caused by a combination of genetic, environmental, and other factors, featured on elevated diastolic and/or systolic blood pressure. Chronically elevated blood pressure can lead to a gradual thickening of the aortic wall, dilation of the lumen, and eventually changes in the composition of the vessel wall and vascular calcification. In turn, vascular calcification might reduce the elasticity of artery walls, increase artery stiffness, and further increase blood pressure, forming a vicious cycle (25). The polymorphism in the gene encoding irisin (FNDC5 rs1746661) has been identified to be related to high systolic blood pressure and dyslipidemia in female patients with type 2 diabetes (T2DM) (26). Besides, a negative correlation has been shown between circulating irisin and blood pressure. Irisin levels decreased as blood pressure increased in preeclampsia and dialysis patients (27, 28). Compared to non-hypertensive diabetics, serum irisin is negatively correlated with blood pressure in hypertensive patients (29). All the above evidence indicates that irisin is significantly related to hypertension, and may be involved in the regulation of blood pressure.

Endothelial dysfunction, vascular inflammation, and vascular remodeling lead to increased peripheral resistance and vascular damage, which accelerates the rise in blood pressure (30). The subsequent mechanistic study showed that irisin supplementation could improve mesenteric artery endothelial dysfunction and reduce blood pressure in spontaneously hypertensive rats through AMPK-AKT-NO signaling pathway (31). Irisin could lower blood pressure by inhibiting oxidative stress and inflammation in Zucker diabetic adipose rats (32). Irisin might alleviate endoplasmic reticulum stress of vascular smooth muscle cells (VSMCs) by activating AMPK and inhibiting p38 signaling pathway, thereby protecting hypertension and vascular remodeling (33). Fortunately, irisin happens to be a multifunctional factor, acting on the above-mentioned pathophysiology and thus participating in the regulation of blood pressure.

Interestingly, treatment of amlodipine and valsartan (12 weeks) increased irisin levels in hypertensive patients (34). In addition, intravenous irisin effectively reduced blood pressure via Nrf2-mediated antioxidant effects in the paraventricular nucleus of spontaneously hypertensive rats (35). However, irisin might increase blood pressure and cardiac contractility when it was injected into the third ventricle of rats by activating neurons in the paraventricular nucleus of the hypothalamus (36). It implies that central and peripheral irisin may have different regulatory effects on blood pressure, and the specific mechanism still needs to be further explored.

Most of the above evidence suggests that irisin has a protective effect against hypertension. However, in Nω-Nitro-L-arginine methyl ester hydrochloride-induced models of hypertension, chronic irisin treatment with physiological doses did not reduce blood pressure (37). An observational study has also shown that irisin levels are positively correlated with systolic blood pressure and could serve as an independent predictor of hypertension. Subgroup analysis implied that irisin was associated with hypertension-related stroke significantly (38). The controversial results may be caused by different hypertension populations and different animal models of hypertension. In addition, previous studies have shown that central and peripheral irisin may have different regulation of blood pressure, and central irisin supplementation may lead to increased blood pressure, which may explain the correlation between irisin and hypertensive stroke. In the future, the effects of irisin should be evaluated separately for specific populations and different modes of administration and dosages.

### Irisin and diabetes

Diabetes mellitus is a multi-etiological metabolic disease caused by insufficient insulin secretion and/or defective function, which is also a major cause of vascular calcification. A meta-analysis showed that the percentages of coronary artery calcium score (CACS)>0, CACS≥100, and CACS≥400 in diabetes patients were 29.3%-86.0%, 22.8%-65.0%, and 7.0%-37.8%, respectively (39). The PREDICT study suggested that CACS was a strong predictor of MACE in asymptomatic T2DM patients, with CACS 101-1000 patients having a 10.5-fold increased risk and CACS exceeding 1000 patients having a 19.8-fold increased risk of cardiovascular events (40). Obviously, diabetes is a major risk factor for vascular calcification.

It has been proved that irisin might regulate glucose metabolism, promote glucose utilization, and improve blood glucose disorders (41). Compared with healthy people, T2DM patients had lower circulating irisin levels, while the circulating irisin levels of long-standing T1DM patients and newly diagnosed T2DM patients were higher than that of the control group (42–47). Even, in patients with diabetic complications including diabetic retinopathy and diabetic nephropathy, irisin levels may be further reduced (48, 49). Therefore, irisin may be a promising predictor of diabetes’ development and progression. As for the elevated irisin levels in patients with new onset T2DM, a protective increase may be one of the reasons. The inconsistency in patients with different types of diabetes may be related to the different etiology of the two types of diabetes and the complexity of the mechanism of irisin regulating homeostasis.

Insulin resistance and abnormal pancreatic function are common pathological features of T2DM. A meta-analysis enrolling non-diabetic patients confirmed that circulating irisin was associated with insulin resistance (48). And irisin can increase energy expenditure, reduce body weight, and improve insulin sensitivity (50, 51). For example, irisin ameliorated insulin resistance by preserving the AMPK-insulin receptor signaling pathway in myoblasts (52). Pancreatic β-cells dysfunction is another common cause of diabetes, and chronic damage to pancreatic β-cells can further increase insulin resistance. Irisin can reduce the apoptosis of pancreatic β-cells by regulating apoptosis-related proteins (53). Both *in vivo* and *in vitro* studies have confirmed that irisin can promote the proliferation of pancreatic β-cells, increase insulin secretion and improve the function of pancreatic β-cells (53, 54). Additionally, irisin could alleviate insulin resistance and inflammation in pancreatic β-cells by activating PI3K/AKT/FOXO1 signaling pathway and inhibiting TLR4/NF-κB signaling pathway (55). In addition to the above effect, irisin promoted GLUT4 translocation and glucose uptake in skeletal muscle through AMPK signaling pathway and MAPK signaling pathway (56–58). Irisin could induce fat browning, increase insulin sensitivity and increase glucose uptake in adipose tissue (59–61). Studies have also shown that irisin can regulate hepatic glycogen synthesis and gluconeogenesis through multiple signaling pathways to maintain glucose homeostasis (62, 63).

Current results confirm that irisin plays a multi-dimensional protective role in diabetes by reducing insulin resistance, promoting β cell proliferation, increasing insulin secretion, improving glucose uptake in peripheral tissues, and regulating gluconeogenesis.

### Irisin and CKD

There are many risk factors for vascular calcification in CKD patients, including hyperphosphatemia, hypercalcemia, increased mineralization, uremic toxins, and hemodynamic abnormalities caused by hemodialysis (64). Irisin was significantly decreased in CKD patients and its decreased levels are negatively associated with blood urea nitrogen and creatinine (65–69). For dialysis patients, the irisin levels of peritoneal dialysis patients were higher than those of hemodialysis patients, and the levels were related to the adequacy of peritoneal dialysis (70, 71). In addition, low serum irisin levels were associated with significantly increased risks of vascular calcification, cardiovascular disease, and cardiovascular and cerebrovascular disease mortality in hemodialysis patients (72–74). Also, irisin could independently predict carotid atherosclerosis and sarcopenia in peritoneal dialysis patients (75). Collectively, low irisin in CKD patients leads to loss of muscle as well as the occurrence and death of cardiovascular diseases.

Mechanistically, chronic renal failure can induce skeletal muscle atrophy in mice by inhibiting FNDC5 expression and enhancing skeletal muscle autophagy (76). Renal failure can inhibit irisin in muscle and cause the loss of cortical bone mass in mice (77). Peng et al. found that irisin can improve energy metabolism and reduce kidney injury and fibrosis (78). Mechanistically, muscle-specific overexpression of PGC-1α could increase the production of irisin in three models of kidney cell damage. In addition, irisin might correct metabolic reprogramming, increase the production of ATP in damaged kidney cells, and inhibit progressive kidney injury and fibrosis. Dojuksan can improve renal fibrosis through irisin-mediated muscle-kidney crosstalk (79). Furthermore, irisin has also been identified to prevent obesity-related CKD by regulating perirenal adipose tissue function in obese mice (80, 81). Irisin can improve CKD and its complications by regulating muscle-kidney and fat-kidney interactions. Unfortunately, as the typic feature of CKD, the correlation between calcium and phosphorus metabolism disorders and irisin has been poorly studied, which should be the focus of future attention.

### Irisin and inflammation

Inflammation plays a critical role in the pathogenesis of vascular calcification. Macrophages or T lymphocytes infiltrating blood vessels are stimulated to produce TNF-α, IL-1β, IL-6, and other pro-inflammatory factors involved in vascular calcification (82, 83). Irisin could lower several inflammatory cytokines and increase anti-inflammatory factors (84). Irisin can reduce inflammatory markers such as MCP1, ICAM1, and VCAM1 in atherosclerotic APOE^-/-^ mice (59). In mechanism, anti-inflammatory properties of irisin are connected with the reduced activity of NF-κB or AMPK signaling pathway activation (85, 86). Irisin could polarize M0 and M1 macrophages towards M2 phenotype by activating AMPK signaling (87). Besides, irisin could promote the differentiation of M2 macrophages by inducing the transcriptional activation of the JAK2-Stat6-dependent PPAR-gamma-associated anti-inflammatory system and NRF2-associated antioxidant genes (88). In addition, irisin alleviates inflammation by restraining the NLRP3 inflammasome and NF-κB pathway (89). Irisin can inhibit the formation of neutrophil extracellular trap and protect the pancreatic injury of mice, further elucidating the protective effect of irisin on acute inflammatory injury (90). Taken together, irisin plays an important role in the resistance to inflammation signaling pathway, providing a theoretical basis for its clinical transformation.

### Irisin and oxidative stress

Oxidative stress can further induce mitochondrial dysfunction, endoplasmic reticulum stress, and lysosome dysfunction, leading to vascular calcification (91). At the molecular level, oxidative stress promotes the expression of MSX2, Runx2, SOX9, and ALP in cells and causes vascular calcification (92). In addition, oxidative stress facilitates the occurrence and development of vascular calcification through NADPH oxidase and its downstream ERK signaling pathway (93). By activating Nrf2/HO-1 signaling pathway, irisin could increase the expression of key antioxidant enzymes, such as catalase-9, superoxide dismutase, and glutathione peroxidase, and inhibit the production of hydrogen peroxide, so as to protect vascular endothelial cells from cellular oxidative damage (94). Irisin has been clearly confirmed to have an antioxidant function, and the antioxidant mechanism of irisin is associated with important cellular processes by reducing ROS and its complications, including regulation of mitochondrial fission and fusion, inhibition of inflammasome activation, improvement of autophagy, inhibition of endoplasmic reticulum stress and ferroptosis, and reversal of cell death (95).

## Irisin inhibits vascular calcification

**Figure 2:**
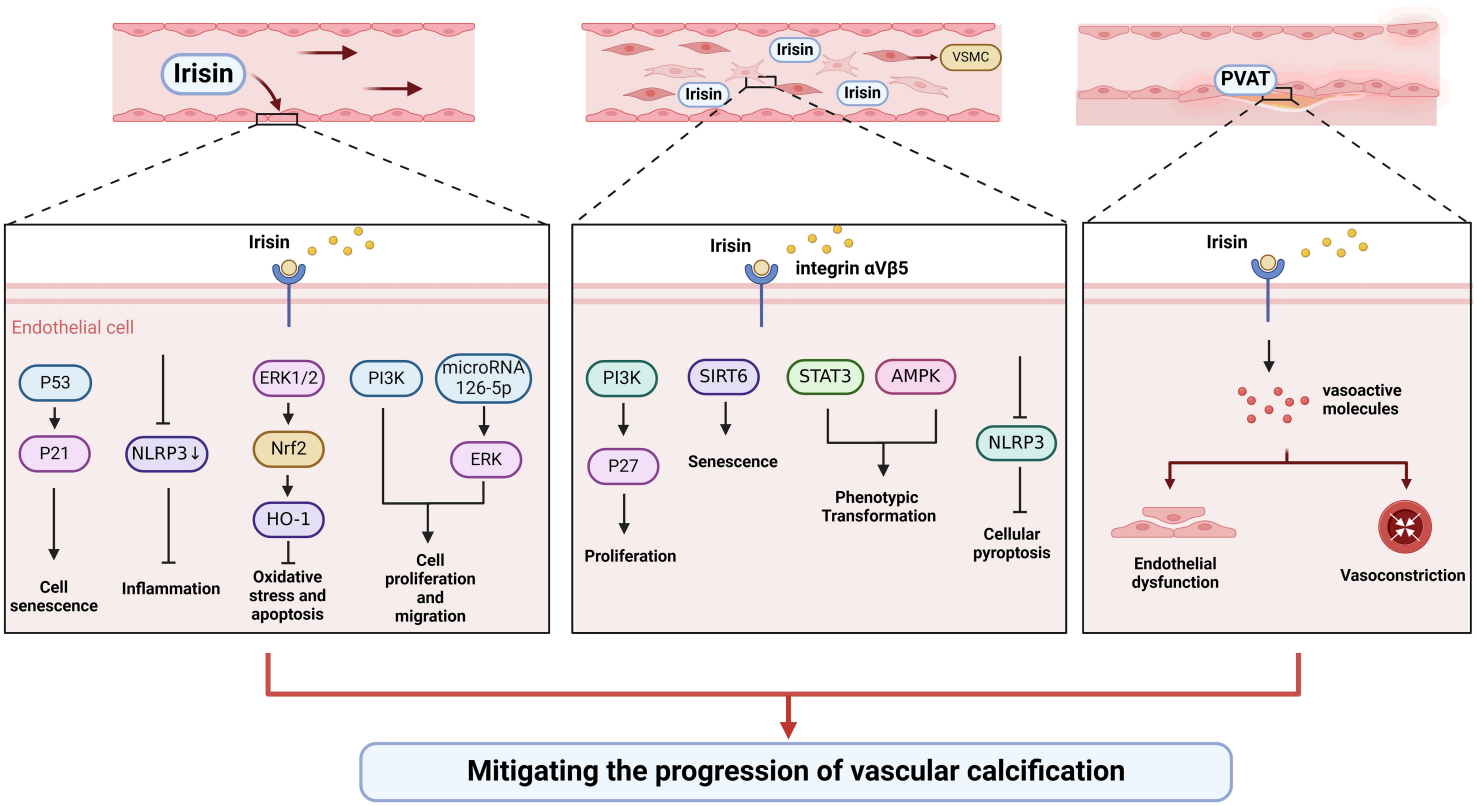
Irisin could alleviate vascular calcification by regulating endothelial cell function and phenotypic switching of VSMCs.

In a narrow sense, vascular calcification can be divided into three types according to the location: intima calcification, media calcification, and calcification of the extravascular membrane. Intima calcification is closely associated primarily with atherosclerotic plaque, while media calcification is considered to be the dominant form of vascular calcification in patients with CKD and hypertension (95). Multiple stimuli have been identified to induce endothelial cell dysfunction and VSMCs phenotypic transformation, leading to the development of vascular calcification (96). Previous studies have shown that irisin can relieve vascular calcification by acting on stem cells, vascular endothelial cells, and VSMCs (**Table 1**, **Figure 2**).

**Table 1:** The protective effect of irisin in vascular calcification.

Author	Cell type	Animal Model	Signaling pathways	Pathophysiological process	Reference
Zhu	EPCs	Diabetes Mellitus Mice	PI3K/Akt pathway	EPCs proliferation	(97)
Deng	HUVEC	/	ROS-NLRP3 inflammasome signaling	Inhibition of inflammation	(98)
Xin	CMEC	/	NLRP3 inflammasome	Inhibition of NLRP3 activation	(99)
Zhu	HUVEC	Type 2 diabetic mice	PKC-β/NADPH oxidase and NF-κB/iNOS pathways	Inhibition of oxidative/nitrification stress	(100)
Zhu	CMEC	Diabetic mice	ERK1/2/Nrf2/HO-1 pathway	Inhibition of Oxidative Stress	(94)
Pan	CMEC	Doxorubicin -induced cardiotoxicity mouse model	NF-κB-Snail pathway	Inhibition of endothelial cell mesenchymal transformation	(101)
Chen	HUVEC	Nicotine-Mediated mice	integrin αVβ5/PI3K pathway and P53/P21 pathway	Inhibition of HUVEC proliferation, migration and senescence	(102)
Bi	HMEC, HUVEC	Microvascular leakage murine model	MLCK–β-catenin pathway and AMPK-Cdc42/Rac1 pathway	Enhancement of endothelial junctions and barrier function	(103)
Zhang	HUVEC	A carotid partial ligation model of apolipoprotein E-deficient mice	microRNA126-5p-ERK signaling pathway	Enhancement of HUVEC survival and proliferation	(104)
Liao	HUVEC	Myocardial infarction Mice	ERK signaling pathway	Enhancement of the HUVEC migration and angiogenesis	(105)
Li	/	Nicotine-mediated atherosclerosis	integrin αVβ5/PI3K/P27 pathway	Inhibition of VSMCs proliferation	(106)
Chi	VSMCs	Mouse model of aging	FNDC5/irisin-DnaJ/Hsp40-SIRT6 axis	Inhibition of mouse VSMCs senescence	(107)
Song	VSMCs	/	STAT3 signaling pathway	Inhibition of VSMCs phenotype modulation	(108)
Wang	VSMCs	/	AMPK/Drp1 signaling	Inhibition of VSMC osteoblastic transformation	(109)

EPCs, endothelial progenitor cells; HUVEC, human umbilical vein endothelial cell; HMEC, human microvascular endothelial cells; CMEC, cardiac microvascular endothelial cells.

### Irisin and vascular endothelial cells

Endothelial progenitor cells (EPCs) could differentiate and mature into endothelial cells, which are involved in vascular formation and repair after vascular endothelial injury (110). In overweight/obese children, circulating irisin is significantly increased, which is significantly associated with circulating EPCs (111). Exercise and dietary interventions could increase irisin levels and EPCs levels in obese adults (112). In addition, irisin could activate PI3K/Akt pathway and increase the number and function of EPCs in diabetic mice (97). Future studies should focus on how irisin modulates vascular calcification by regulating the number and function of EPCs.

More and more evidence shows that endothelial dysfunction is the primary cause of vascular calcification through the transition to mesenchymal and osteoblast lineages, secretion of calcification growth factors, induction of endothelial alkaline phosphatase (113). Improving the function of the vascular endothelium is essential to slow or reverse vascular calcification. Oxidative stress and inflammation are common causes of endothelial injury. Irisin could inhibit the activation of NLRP3 inflammasome and endothelial dysfunction (98, 99). Besides, irisin could improve endothelial function in T2DM by inhibiting oxidative/nitrifying stress (100). Irisin can protect diabetic myocardial microvascular endothelial cells from apoptosis and oxidative stress through ERK1/2/Nrf2/HO-1 pathway (94). In addition to being anti-inflammatory and antioxidant, irisin also inhibits endothelial cell mesenchymal transformation by regulating endothelial cell ROS accumulation and autophagy disorders, thus improving vascular fibrosis (101). Irisin antagonizes vascular endothelial cell proliferation and migration through the integrin αVβ5/PI3K pathway and microRNA126-5p-ERK signaling pathway (102–104). Besides, irisin inhibited endothelial cell senescence and G0/G1 phase cell cycle arrest through the p53/p21 pathway (102). Also, irisin might enhance the angiogenesis of mesenchymal stem cells and human umbilical vein endothelial cells (105, 114–117). Therefore, irisin can protect vascular endothelium in multiple directions.

### Irisin and VSMCs

VSMCs are the most abundant cell types, mainly located in the media, and their senescence, apoptosis, and phenotypic transformation have been confirmed to be the main causes of the development of vascular calcification (64). A histological study showed that irisin could increase the intima-media thickness, the number of VSMCs, and elastic layers in the media of the thoracic aorta (118). Irisin might inhibit vascular thickening and VSMCs proliferation through the integrin αVβ5/PI3K/P27 pathway (106). In addition, irisin can reduce the senescence of mouse VSMCs by increasing the stability of SIRT6, and irisin-rich extracellular vesicles can be used to delay vascular aging (107). As a typical feature of vascular calcification, irisin may be involved in the phenotypic transformation of VSMCs. For instance, irisin reversed PDGF-BB-induced VSMCs to secretory transformation through the STAT3 signaling pathway (108). Furthermore, irisin alleviated vascular calcification by activating autophagy and inhibiting NLRP3-mediated VSMCs pyroptosis in CKD (109). Irisin inhibited VSMC osteoblast transformation and mitochondrial dysfunction through AMPK/Drp1 signaling pathway to alleviate vascular calcification in CKD (109). Taken together, irisin might participate in the regulation of VSMCs proliferation, senescence, and phenotypic transformation to delay vascular calcification.

## Discussion

Vascular calcification is one of the most difficult problems in cardiovascular diseases, and it is urgent for us to explore effective treatments to reduce adverse events and death and alleviate the burden of disease. Irisin is a so-called “exercise hormone” that is secreted into the circulation in response to stimulation such as physical exercise (119). Current evidence suggests that irisin can protect against hypertension by regulating endothelial function, inflammatory oxidative stress. In addition, irisin can improve insulin resistance, enhance islet function, and promote sugar utilization to optimize blood glucose levels. It also can modify kidney energy metabolism and adipose tissue function to protect kidney function. As a multifunctional cytokine, irisin can be involved in the inhibition of risk factors for vascular calcification through multiple pathways.

Circulating irisin has been shown to be significantly associated with hypertension, diabetes, CKD, and other risk factors for vascular calcification. Moreover, lower serum irisin levels are in connection with a higher prevalence and progression of vascular calcification, including abdominal aortic calcification, coronary artery calcification, and aortic valve calcification (72, 120–122). Therefore, irisin might be used as an indicator of risk factors and vascular calcification. However, more retrospective studies and cohort studies should be performed to confirm its ability and value as a marker.

In addition to being used as an indicator, irisin therapy can also improve hypertension, insulin resistance, etc. While most studies have shown a protective effect of irisin, there are also studies that suggest the opposite. For example, different parts of the injection of irisin have opposite effects on blood pressure. Therefore, the administration site and dose of irisin are worthy of further study. As an exercise-related cytokine, exercise can increase circulating irisin, but it is still unknown whether and how it reaches the lesion site to relieve vascular calcification.

Endothelial dysfunction and phenotypic transformation of VSMCs are two characteristics of vascular calcification. Irisin can directly protect endothelial function by regulating endothelial cell proliferation, senescence, interstitial transformation, and angiogenesis. And irisin can be used to defend against vascular calcification by regulating the proliferation, senescence, pyroptosis, and osteogenesis transformation of VSMCs. Besides, irisin has been identified to improve insulin sensitivity, inhibit bone resorption, and relieve bone-vascular interactions, thereby protecting vascular function (123). These studies elucidate the molecular mechanism of irisin to alleviate vascular calcification. In the future, the relationship between irisin and endothelial cells-VSMCs crosstalk is worth exploring.

Perivascular adipose tissue (PVAT) is a key endocrine organ around blood vessels that could secrete a large amount of metabolic vasoactive factors by endocrine and paracrine means (124). Under physiological conditions, PVAT exerts an anti-vasoconstriction effect by releasing many vasoactive molecules (125). Accumulating evidence points towards that obesity-driven adipose tissue dysfunction promotes chronic inflammatory states within the body, thereby contributing to the pathogenesis of cardiovascular disease (126). Recent research confirmed that irisin can improve the anti-contractile effect and endothelial dysfunction of PVAT in the thoracic aorta of high-fat-fed mice (127, 128). The protective mechanism of irisin may be related to the upregulation of the HO-1/adiponectin axis in PVAT. Therefore, irisin can indirectly mediate the regulation of vascular function by improving the function of PVAT. Furthermore, whether PVAT can secrete irisin to regulate vascular function is also the direction of future research.

## Conclusion

In conclusion, irisin has a predictive and protective effect on vascular calcification and its risk factors. Future large clinical randomized controlled studies should be performed to verify the roles of irisin in vascular calcification.

## Author contributions

SW: Writing – original draft. SH: Writing – original draft. YP: Writing – original draft, Writing – review & editing.
